# Preparation and Enrichment Properties of Magnetic Dodecyl Chitosan/Silica Composite for Emerging Bisphenol Contaminants

**DOI:** 10.3390/ma11101881

**Published:** 2018-10-02

**Authors:** Jingrong Hu, Wangwei Liu, Huiling Liu, Lamei Wu, Huijuan Zhang

**Affiliations:** 1 Key Laboratory of Analytical Chemistry of the State Ethnic Affairs Commission, College of Chemistry and Material Science, South-Central University for Nationalities, Wuhan 430074, China; ahrr123456@163.com (J.H.); yieyie0506@163.com (W.L.); 2 Experimental Teaching and Laboratory Management Center, South-Central University for Nationalities, Wuhan 430074, China; znmzdx_ms@sina.com; 3 Key Laboratory of Analytical Chemistry for Biology and Medicine (Ministry of Education), Department of Chemistry, Wuhan University, Wuhan 430072, China

**Keywords:** magnetic dodecyl chitosan/silica composite, bisphenols, enrichment

## Abstract

Magnetic dodecyl chitosan/silica composite was synthesized and characterized for the enrichment of emerging bisphenol contaminants. The result demonstrated that bispehnol A, bisphenol AF, bisphenol F, and bisphenol S can be efficiently extracted via the resulting composite from aqueous solution. The adsorption rate of four investigated bisphenols on the resulting composite was achieved in the range of 87–99% within 15 min. Bispehnol A was taken as a representative adsorbate to investigate the adsorption studies in detail. The hydrophobic interaction was proposed as the principal mechanism for the adsorption of BPs. The satisfactory reusability of the resulting composite can be quickly achieved by magnetic separation technologies. Magnetic dodecyl chitosan/silica composite has a potential to be applied as a type of efficient and easily recyclable sorbent for the removal of trace organic pollutants from aqueous sample.

## 1. Introduction

In past decades, bisphenol A (BPA) as the most used bisphenol chemical in industrial fields have been frequently pointed out to possess endocrine disruptive activities even at low concentration and potential pathogenic risk [1,2]. In order to avoid the adverse effects of BPA exposure on health, the applications of BPA are strictly controlled in the manufacture of food-related containers and packages, especially those involved in products of infants and pregnant women [3,4,5]. Some BPA analogues such as bisphenol S (BPS), bisphenol F (BPF), and bisphenol B (BPB) have been synthesized and tried to replace BPA in the industrial production. Unfortunately, some toxicological studies showed that these BPA analogues also induce similar or stronger harmful responses in comparison with BPA [6,7,8]. BPA and its analogues can enter into the natural environment through a variety of anthropogenic activities due to the incomplete purification of wastewater treatment. Bisphenols (BPs) has been found at trace amount levels in surface water, sediment, and indoor dust [9,10]. Moreover, BPs can be ingested directly through the foods or beverages that have been contaminated from plastic containers and packages [11,12,13]. Therefore, it is essential for reducing human exposure to decontaminate micro-level emerging bisphenol contaminants in food and environmental matrices.

Sorption technique is deemed to be promising for the removal of contaminants from aqueous systems owing to its relatively low cost, ease of implementation, and high efficiency. The efficiency of the sorption technique depended mainly on the sorption performance of the sorbent. Recently, extensive efforts have been taken for the development of sustainable efficient sorbents with increasing concern on the environmental impact of petroleum-based materials [14,15]. Chitosan (CTS), as a natural environment-friendly biopolymer, has displayed unique superiority in the removal of metal ions and charged dyes because of the presence of amino and hydroxyl groups in the structure [16,17,18]. However, unsatisfying mechanical properties and the lack of the hydrophobic functional structure limited the application of chitosan in organic contaminant treatment. Improving the properties of chitosan materials is still the major challenge in expanding their application in the contaminant treatment. 

Compared with the self-cross-linking, more researchers focused on compositing chitosan with other distinctive materials like bentonite [19], active charcoal [20], and polymer [21]. Among various chitosan composites, the compositing of chitosan with silica can improve more efficiently the phy-chemical properties of chitosan-based composites including the mechanical stability, porous structure, specific surface area, as well as high biocompatibility [22,23]. Various silicon alkoxide with active groups can be chosen as the precursors of silica components to modify the characteristic of chitosan/silica composites [24]. The homogeneous structure of the chitosan/silica composite is expected to be obtained more easily than other inorganic matrices through the sol-gel method [25]. On the other hand, the separation mode and regeneration of the sorbents from the sample matrix also affect the popularization of sorption technique in the real application. Magnetic materials have come into researchers’ notice because they can be more easily separated and recycled from aqueous media via external magnetic fields in comparison of filtration or centrifugation procedure [26]. The sorption efficiency can be enhanced by improving the mass transfer of the analytes between magnetic sorbents and sample solution. Meanwhile, the coating can not only improve their dispersibility but also protect magnetic activity of the magnetic core from the complex matrices. Currently, the design and preparations of magnetic chitosan-based sorbents were still focused on the uptake of harmful metal ions or organic dyes [27,28,29]. Not many studies on chitosan-based sorbents for the removal of emerging BPs contaminants are reported [30,31].

Thus, the aim of this study was to prepare magnetic dodecyl chitosan/silica composite and investigate the enrichment behavior of emerging BP contaminants on the resulting materials. The adsorption efficiency of the resulting composite was evaluated through four BPs including BPS, BPF, BPAF, and BPA. In order to understand the probable adsorption mechanism, BPA was taken as a representative adsorbate for detailed adsorption studies. Furthermore, the enrichment ability and reusability of the composites were investigated as well. 

## 2. Materials and Methods

### 2.1. Materials and Reagents

Fe_3_O_4_ nanoparticles (average particle size is about 100 nm), chitosan (CTS, 80–95% degree of deacetylation, BR), polyvinylpyrrolidone (PVP, K-30, GR), sodium borohydride (NaBH_4_, 96.0%), acetic acid (HAc, 99.5%), ammonium hydroxide (NH_3_·H_2_O, 25%), glutaraldehyde solution (GA, 25 wt. %), sodium hydroxide (NaOH, 96.0%), phosphoric acid (H_3_PO_4_, 85%), ether (99.5%), anhydrous ethanol (99.7%), liquid paraffin (CP) and Span 80 (CP) were purchased from Sinopharm Chemical Reagent Co., Ltd. (Shanghai, China). HPLC grade acetonitrile and methanol were purchased from Tedia Company, Inc. (Fairfield, OH, USA). Tetraethoxysilane (TEOS, >99%), Bisphenol S (BPS, 99%), Bisphenol F (BPF, 99.0%), Bisphenol AF (BPAF, 98%), and Bisphenol A (BPA, 99.0%) and n-dodecanal (95%) were purchased from Aladdin Industrial Corporation (Shanghai, China). The distilled (DI) water used in all the experiments was purified by a Molelement 1815a system (Molecular Technology Instrument Co. Ltd., Shanghai, China).

The stock solution of BPs (0.1 g/L) was prepared by dissolving the corresponding pure chemical in methanol. A stock solution of phosphate buffer solutions (PBS, 1.0 mol/L) was prepared by dissolving in DI water accurate amounts of H_3_PO_4_. The PBS stock solution with different pH value was adjusted with 5 mol/L of NaOH solution. All stock solutions were stored at 4 °C. The working buffer solutions and standard BPs solutions were prepared by diluting the above stock solutions with DI water. 

### 2.2. Preparation of Magnetic Dodecyl Chitosan/Silica Composite

Firstly, magnetic silica microspheres (Fe_3_O_4_@SiO_2_) were synthesized by sol-gel process. The as-prepared Fe_3_O_4_@SiO_2_ microsphere was washed fully with distilled water and anhydrous ethanol, and then dried overnight under vacuum at 50 °C. Secondly, magnetic chitosan/silica composite (Fe_3_O_4_@CTS/SiO_2_) was prepared by inverse suspension polymerization and sol-gel method. The procedure was described as follows: 0.3 g Fe_3_O_4_@SiO_2_ microsphere, 1.2 mL of TEOS, 1 g of chitosan and 0.5 g of PVP were mixed with 50mL HAc solution (2 wt. %). The mixtures were continuously stirred for 12 h at room temperature to obtain a homogeneous suspension. After that, this suspension was added slowly to a solution with 80 mL of liquid paraffin and 2 mL of span-80, and then kept stirring for 2 h at 60 °C. Subsequently, 2 mL of NH_3_·H_2_O and 0.1 mL of GA was added dropwise and the reaction was kept for 1 h. When the reaction was finished, ether was used to demulsify the suspension. The products were collected and washed fully with anhydrous ethanol. Finally, 0.1 g Fe_3_O_4_@CTS/SiO_2_ composite and 0.18 mL n-dodecanal were reacted in 15 mL methanol at 30°C for 6 h. After that, 0.3 g NaBH_4_ was added in the mixture and stirred at 30°C overnight. Magnetic dodecyl chitosan/silica composite (Fe_3_O_4_@CTS/SiO_2_-C_12_) was washed fully with anhydrous ethanol, followed by dried under vacuum at 50 °C.

### 2.3. Characterization Methods

The functional groups of Fe_3_O_4_ nanoparticle, Fe_3_O_4_@SiO_2_ microsphere, Fe_3_O_4_@CTS/SiO_2_ composite and Fe_3_O_4_@CTS/SiO_2_-C_12_ composite were identified in the range from 500 to 4000 cm^−1^ by Fourier Transform Infrared Spectroscopy (FTIR) spectrometer (NEXUS 470, Thermo Fisher Scientific, Waltham, MA, USA). X-ray diffraction (XRD) patterns of the relevant materials were obtained by using a diffractometer with equipped Cu K_α_ radiation (D8 ADVANCE, Bruker, Karlsruhe, Germany). The scanning speed was set at 10 °/min in the region of the diffraction angle from 10° to 80°. Thermal stability of the prepared composite was determined by thermogravimetric analyzer (Diamond TG/DTA 6300, Perkin Elmer, Waltham, MA, USA), which was heated from 30 to 950 °C at a heating rate of 10 °C/min under nitrogen atmosphere. Nitrogen adsorption-desorption isotherms were measured on a surface area and pore size analyzer (SA 3100, Bechman Coulter, Brea, CA, USA) in the range of relative pressures P_s_/P_0_ from 0 to 0.9877. The specific surface area and pore volume were calculated by the Brunauer-Emmett-Teller (BET) method, and the pore size distribution was obtained by using the Barrett-Joyner-Halenda (BJH) theory. 

### 2.4. Adsorption and Desorption Experiments

#### 2.4.1. Batch Adsorption Experiments

Batch adsorption experiments were generally carried out by agitating the PTFE centrifuge tubes containing a certain amount of Fe_3_O_4_@CTS/SiO_2_-C_12_ composite and 10.00 mL of BPs solution on a vortex mixer at 25 °C. The working Fe_3_O_4_@CTS/SiO_2_-C_12_ composites were pre-activated successively by 5.0 mL methanol and 5.0 mL DI water. Each activated procedure was kept for 10 min. After a certain time of vortex, the residual concentration of BPs solution was determined from a constructed calibration curve. The adsorption rate (*A.R.* (*%*)), adsorption capacity at any time (*q_t_*, mg/g) and equilibrium adsorption capacity (*q_e_*, mg/g) were calculated as following equations (Equations (1)–(3)), respectively:

(1) $$A.R.(\%)=\frac{c_{0}-c_{t}}{c_{0}}\times 100\%,$$

(2) $$q_{t}=\frac{c_{0}-c_{t}}{m}V_{0},$$

(3) $$q_{e}=\frac{c_{0}-c_{e}}{m}V_{0},$$

where *c*_0_ (mg/L) is the initial concentration of BPA, *c_t_* (mg/L) is the BPA concentration at any time (*t*, min), *c_e_* (mg/L) is the equilibrium BPA concentration in aqueous phase, *V*_0_ (L) is the initial volume of the BPA solution, and *m* (g) is the amount of the adsorbents.

#### 2.4.2. Desorption and Regeneration Studies

After finishing the adsorption procedure, the BPs-loaded composites were collected from the solution by the magnet and washed with 5 mL DI water to remove the un-loaded BPs. The residual DI water in the composites was dried by nitrogen gas. Finally, the BPs-loaded composites were eluted with a certain amount of eluent in similar manner for 10 min. Then, the composites were separated from the eluent under the magnetic field. The eluent was collected and analyzed. The used composites were reactivated again for reuse. In order to evaluate the reusability of the composites, this adsorption-desorption cycle was repeated for 9 times. 

The desorption performance of Fe_3_O_4_@CTS/SiO_2_-C_12_ composite was expressed by desorption rate, *D.R. (*%*)*, of BPs, and was calculated as following equation (Equation (4)):

(4) $$D.R.(\%)=\frac{W}{W_{0}}\times 100\%,$$

where *W* (mg) is the mass of BPs in the eluent, and *W*_0_ (mg) is the initial mass of BPs. 

### 2.5. HPLC Analysis 

An Agilent 1260 HPLC-DAD system equipped with a WondaSilTM C18 column (Shimadzu, Kyoto, Japan, 150 mm × 4.6 mm, 5 μm) was used for separation and detection of BPs. A gradient elution was adopted by combining solvent A (acetonitrile) and solvent B (water) as follows: at the beginning of the gradient elution, the mobile phase was set as 40% of solvent A, and then the content of solvent A was changed from 40% to 80% within 5 min. Subsequently, the content of solvent A was kept at 80% for 5 min. In all analysis, the column temperature was always kept at 30 °C. The flow rate was kept at 1.0 mL/min. The detection wavelength was set at 225 nm. The injection volume was 10 μL for each analysis.

## 3. Results and Discussion

### 3.1. Characterization 

**Figure 1:**
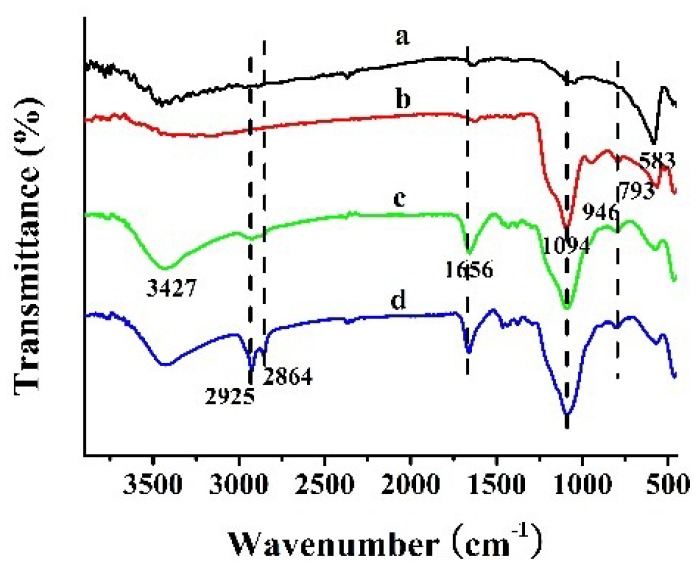
FTIR spectra of (**a**) Fe_3_O_4_; (**b**)Fe_3_O_4_@SiO_2_; (**c**)Fe_3_O_4_@CTS/SiO_2_ composite; and (**d**) Fe_3_O_4_@CTS/SiO_2_-C_12_ composite.

FTIR spectroscopy was used to elaborate the functional groups of Fe_3_O_4_@CTS/SiO_2_-C_12_ composite, as shown in Figure 1. The typical absorption peak for Fe_3_O_4_ nanoparticles (Figure 1a) at 583 cm^−1^ was attributed to Fe–O stretching vibration [20]. This band can be also observed in the FTIR spectrums of other magnetic composites. The stretching vibrations of N–H bonds at 3427 cm^−1^ and amide band I at 1656 cm^−1^ from chitosan were both appeared in the spectrums of Fe_3_O_4_@CTS/SiO_2_ composite (Figure 1c) and Fe_3_O_4_@CTS/SiO_2_-C_12_ composite (Figure 1d). The presence of bands centered at 793 and 1094 cm^−1^ were most probably due to Si–O vibration, which confirmed the presence of a siliceous network in the composite [23]. Moreover, the intensity of the peaks at 2925 and 2864 cm^−1^ that belonged to the stretching vibrations of C–H bond were obviously increased in the spectrum of Fe_3_O_4_@CTS/SiO_2_-C_12_ composite. The results indicate that dodecyl group has been bonded successfully with Fe_3_O_4_@CTS/SiO_2_ composite.

**Figure 2:**
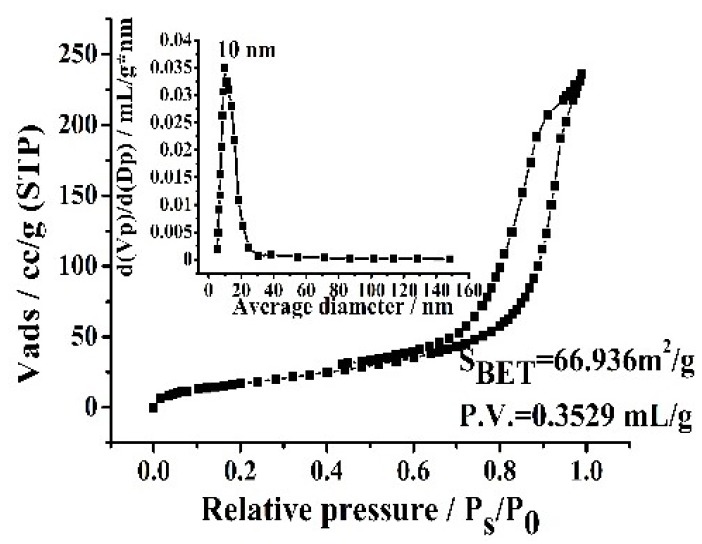
Nitrogen adsorption-desorption isotherms and pore distributions of Fe_3_O_4_@ CTS/SiO_2_-C_12_ composite.

The porosity of Fe_3_O_4_@CTS/SiO_2_-C_12_ composite was investigated by nitrogen adsorption-desorption measurements. As shown in Figure 2, the adsorption-desorption isotherms of the resulting composite belonged to type IV isotherm and the type of hysteresis loop might be classified as type H2 [32]. The pore size can be found to be mainly distributed around 10.0 nm. The surface area (SBET) and total pore volume (*P.V.*) of Fe_3_O_4_@CTS/SiO_2_-C_12_ composite at the optimal synthesis conditions was calculated to be 66.9 m^2^/g and 0.353 mL/g, respectively. 

**Figure 3:**
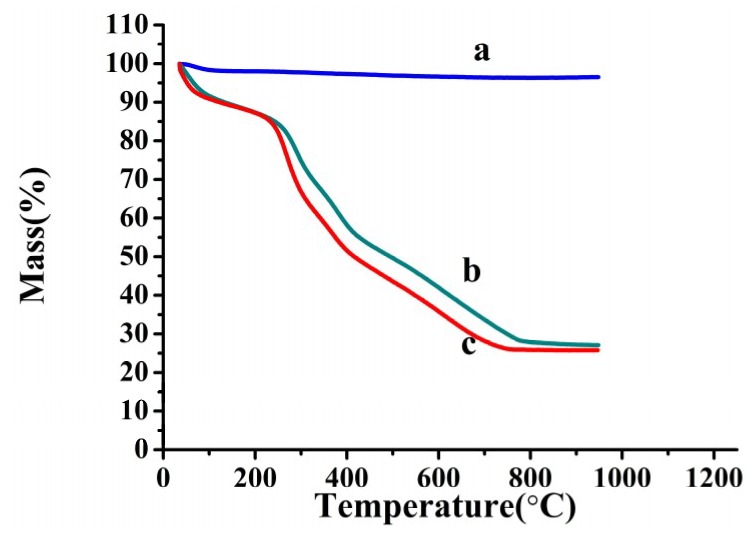
Thermogravimetric profile of: (**a**) Fe_3_O_4_@SiO_2_; (**b**) Fe_3_O_4_@CTS/SiO_2_ composite; and (**c**) Fe_3_O_4_@CTS/SiO_2_-C_12_ composite.

To investigate the thermal stability of Fe_3_O_4_@CTS/SiO_2_-C_12_ composite, the thermogravimetric analysis was performed as shown in Figure 3. 

For three investigated materials, the weight loss on different levels was all occurred from 30 to 112 °C, which might have resulted from the loss of water and gas molecules trapped in the materials. With the increasing of the temperature, a sharp weight loss was started at about 220 °C for magnetic chitosan/silica composite (Figure 3b,c). According to the reported initial decomposition temperature of pure chitosan at near 170 °C [20], it might be attributed to the thermal decompositions of the chitosan component of the composite. It is indicated that the hybridization between chitosan and silica is likely to improve the thermostability of the composite. The thermal decomposition of Fe_3_O_4_@CTS/SiO_2_-C_12_ composite was more rapidly processed in the weight loss region at 220–750°C than that of magnetic dodecyl-free composite. It can be explained that the modification of the dodecyl group resulted in the increasing carbonaceous content of Fe_3_O_4_@CTS/SiO_2_-C_12_ composite. At last, the molecular backbone of the resulting composite was destroyed at 773 °C.

**Figure 4:**
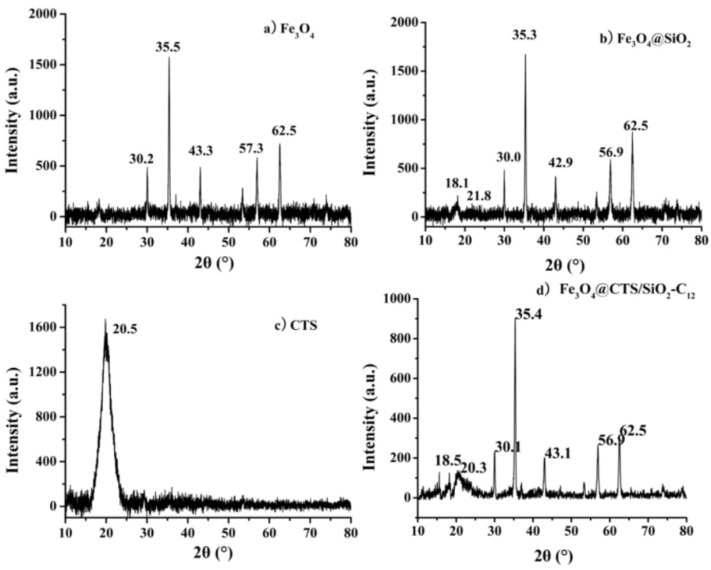
X-ray diffraction (XRD) patterns of: (**a**) Fe_3_O_4_; (**b**) Fe_3_O_4_@SiO_2_ composite; (**c**) CTS; and (**d**) Fe_3_O_4_@CTS/SiO_2_-C_12_ composite.

XRD analysis was conducted to verify the crystallinity of Fe_3_O_4_@CTS/SiO_2_-C_12_ composite. The XRD patterns of the resulting composite and the relevant materials were presented in Figure 4. The characteristic diffraction peaks of Fe_3_O_4_ nanoparticles were at 30.2, 35.5, 43.3, 53.6, 57.3, and 62.5° as shown in Figure 4a. These peaks could be assigned to [220], [311], [400], [422], [511], and [440] planes of magnetite, respectively. As seen in Figure 4b,d, these characteristic diffraction peaks of Fe_3_O_4_ both appeared at the similar position in comparison to the peaks of the raw Fe_3_O_4_ nanoparticles. The result revealed that the surface coating cannot lead to the phase change of the magnetic core. On the other hand, the diminished peak intensity of chitosan at 20.3° and silica at 18.5° in the resulting composite shown in Figure 4d represented that the interaction between chitosan and silica caused an amorphous structure of the coating. 

### 3.2. The Influence of Long Alkyl Group on the Adsorption

**Figure 5:**
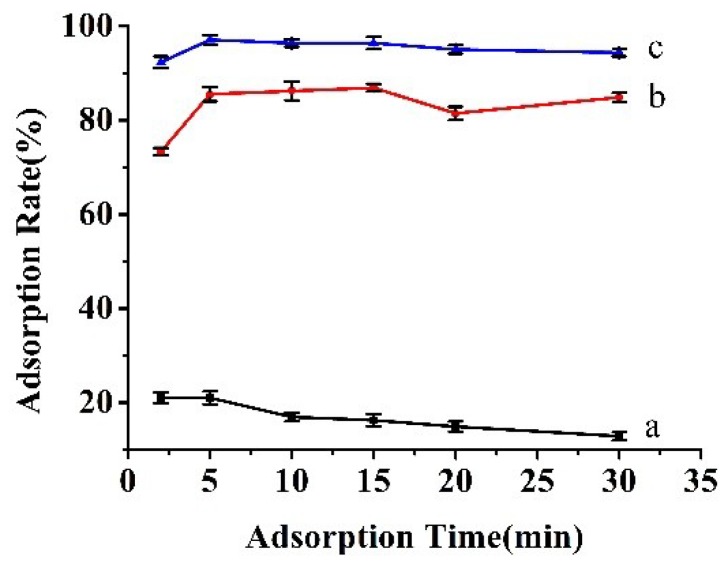
The influence of different alkyl groups on the adsorption. (**a**) Without any modification; (**b**) Octyl modification; (**c**) Dodecyl modification. 50 mg adsorbents in 10.0 mL solution (10 mg/L BPA); 34 mmol/L PBS, pH 7.0; 25 °C. The error bars represent standard deviation of three parallel experiments.

The modification of long alkyl groups may play a crucial role in the adsorption of BPs on the resulting composite. Three magnetic chitosan/silica composites with different alkyl groups were prepared respectively and assessed their adsorption performance for BPA. As seen the result from Figure 5, the adsorption efficiency of the investigated composites are improved markedly through the modification of alkyl group. The highest adsorption efficiency is obtained within 5 min by using the magnetic dodecyl-modified composite. It is suggested that the hydrophobic interaction can dominate the adsorption mechanism between BPA and the Fe_3_O_4_@CTS/SiO_2_-C_12_ composite. 

### 3.3. Influence of pH on Adsorption 

**Figure 6:**
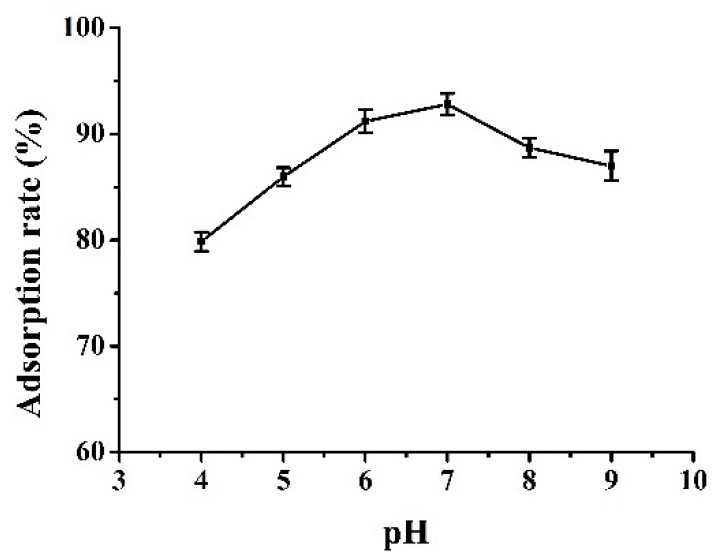
The influence of pH on the adsorption. A mass of 50 mg adsorbents in 10.0 mL solution (10 mg/L BPA); 34 mmol/L PBS; 25 °C. The error bars represent standard deviation of three parallel experiments.

The pH value of solution is an important factor in the adsorption process. The change of pH can affect not only the surface properties of the resulting composite but also charge properties of adsorbate in aqueous phase. From the results presented in Figure 6, the adsorption efficiency of Fe_3_O_4_@CTS/SiO_2_-C_12_ composite for BPA increases gradually in the pH range from 4.0 to 7.0. The maximum adsorption rate of BPA is achieved at pH 7.0. A decreasing tendency of the adsorption efficiency can be observed when the pH value exceeds 7.0. 

According to the reported pK_a_ value of BPA, it ranged from 9.78 to 10.39, BPA presented dominantly the molecular state in the investigated pH range [12]. It is implied that the adsorption mechanism cannot be confined to the hydrophobic interaction mentioned above. The hydrogen bonding between the hydroxyl groups of BPA and the residual amino groups of the composite might also be responsible for the adsorption of BPA. The increasing of pH value strengthens the formation of hydrogen bonding which results in the improvement of the adsorption performance. When the pH value of the solution is close to the reported pK_a_ value of BPA, the partial dissociation of hydroxyl groups of BPA might intensify the repulsive interaction with negative surface charges of the resulting composite. Furthermore, the increasing polarity of BPA also leaded to weaken the hydrophobic interaction between BPA and the composite. Thus, the pH value of the solution was set as 7.0 in the subsequent experiments. 

### 3.4. Adsorption Studies 

#### 3.4.1. Adsorption Kinetics

**Figure 7:**
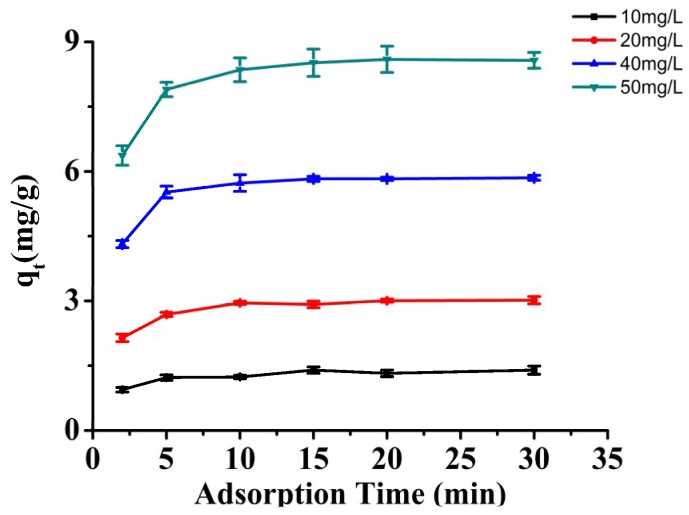
The influence of different initial concentration on the adsorption. A mass of 50 mg adsorbents in 10.0 mL solution; 34 mmol/L PBS, pH 7.0; 25 °C.

The kinetic curves of BPA adsorption on Fe_3_O_4_@CTS+SiO_2_-C_12_ composite were obtained at different initial BPA concentration as shown in Figure 7. The adsorption capacity increased with increasing the initial BPA concentration. A average maximum adsorption capacity of 8.7 mg/g can be reached with the 50 mg/L initial concentration. Furthermore, rapid adsorption was observed and the equilibrium could be attained at around 10 min. It indicated that the adsorption sites of 50 mg prepared composite are abundant for the adsorption even at higher concentrations of BPA.

Lagergren’s pseudo-first-order and pseudo-second-order kinetic models were used to fit the experimental data for understanding the adsorption process of BPA on Fe_3_O_4_@CTS/SiO_2_-C_12_ composite. The Lagergren’s equation for first-order kinetics (Equation (5)) can be written as follows [33]:

(5) $$In(q_{e}-q_{t})=Inq_{e}-k_{1}t.$$

The rate constant *k*_1_ was determined from slope of the linear plot of *In(q_e_* − *q_t_)* against time *t*.

A pseudo-second-order kinetic equation based on adsorption capacity can be expressed in the form (Equation (6)) [34]:

(6) $$\frac{t}{q_{t}}=\frac{1}{k_{2}q_{e}^{2}}+\frac{t}{q_{e}},$$

where *k*_2_ is the rate constant of pseudo-second-order adsorption, which can be calculated from the intercept and slope of the plot of (*t/q_e_*) vs. time *t*.

The obtained kinetic values with correlation coefficients (*R*^2^) were listed in Table 1. The *R^2^* values of pseudo second-order kinetic model at different initial concentration are better (>0.99) than those of pseudo first-order kinetic model, proving that the pseudo second-order kinetics model is more appropriate to explain the adsorption behavior of BPA on Fe_3_O_4_@CTS/SiO_2_-C_12_ composite. 

#### 3.4.2. Adsorption Isotherm

Study of adsorption isotherm is essential to understand the adsorbent-adsorbate interaction. The Langmuir and Freundlich isotherm model were applied to describe the adsorption progress on the resulting composites. These two adsorption isotherm models can be represented as following equations (Equations (7) and (8)), respectively:

(7) $$\frac{c_{e}}{q_{e}}=\frac{c_{e}}{q_{m}}+\frac{1}{K_{L}q_{m}},$$

(8) $$Inq_{e}=InK_{F}+\frac{1}{n}Inc_{e},$$

where *K_L_* (L/mg) is the Langmuir constant, *q_m_* (mg/g) is the maximum adsorption capacity for monolayer formation on adsorbent, *K_F_* ((mg·g)·(mg·L)^−1/n^) is the Freundlich constants, and 1/*n* is a constant depicting the surface heterogeneity of absorbents. 

Similarly, the calculated isotherm parameters with correlation coefficients (*R*^2^) for the adsorption of BPA were summarized in Table 2. The Freundlich isotherm model (*R*^2^ = 0.9947) simulates more properly the adsorption of BPA on the resulting composite compared with the Langmuir one (*R*^2^ = 0.9792). It is demonstrated that the multilayer adsorption behavior carries out on the heterogeneous surface of the composite. Moreover, the value of 1/*n* is less than 1, verifying that the adsorption is favorable.

### 3.5. Evaluation on the Enrichment Performance of the Resulting Composite 

**Figure 8:**
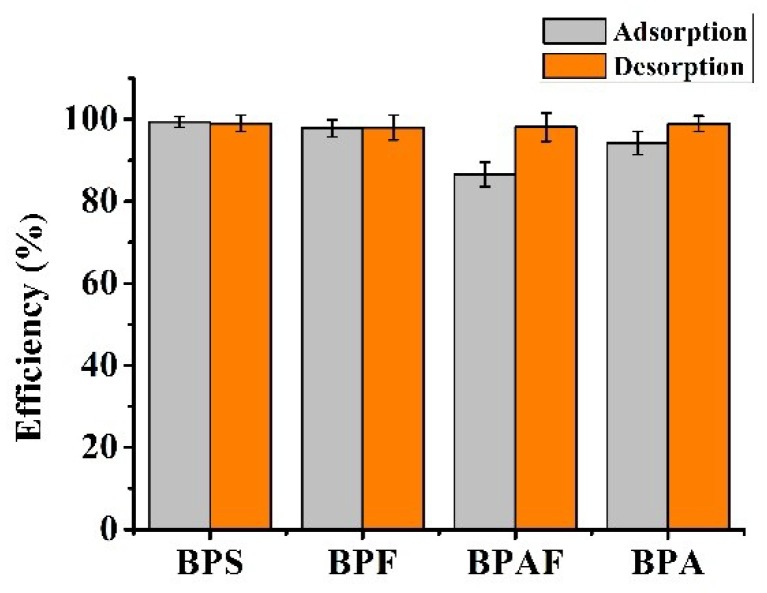
The enrichment performance of BPA, BPS, BPF, and BPAF on Fe_3_O_4_@CTS/SiO_2_-C_12_ composite. 50 mg adsorbents in 10.0 mL solution (each 0.5 mg/L); 34 mmol/L PBS, pH 6.0; 25 °C.

**Figure 9:**
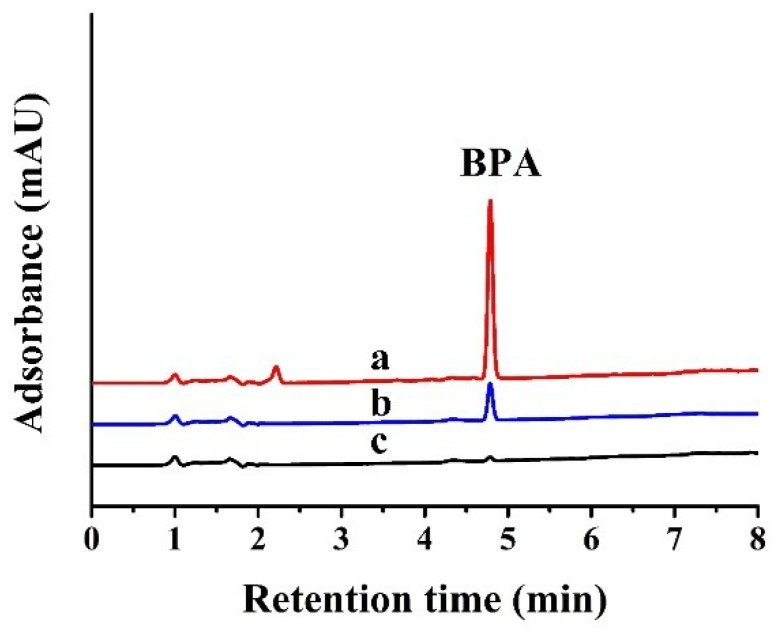
The HPLC-DAD chromatograms of BPA obtained from: (**a**) 2 mL eluent after elution; (**b**) direct injection of the BPA solution; (**c**) the BPA solution after adsorption; 50 mg adsorbents in 10.0 mL solution (1 mg/L BPA) The optimal conditions were used.

Under the optimal conditions, the enrichment performance of Fe_3_O_4_@CTS/SiO_2_-C_12_ composite is evaluated by using the mixed solution of four BPs including BPS, BPF, BPAF, and BPA. Great adsorption of each BPs can be observed apparently in Figure 8. The BPs adsorption efficiency of the resulting composite can be comparable with those of other reported chitosan-based materials [30,31]. Moreover, the similar adsorption efficiency of BPA could be attained more quickly on the resulting composite than commercial chitosan [30]. Considering the hydrophobic interaction between Fe_3_O_4_@CTS+SiO_2_-C_12_ composite and BPs, methanol was chosen as the eluent. The result found that the loaded-BPs can be eluted completely within 10 min by 2 mL of methanol, which means that the resulting composite can be regenerated by small amount eluent washing. The maximum enrichment efficiency of BPS, BPF, BPA, and BPAF are 99%, 97%, 98%, and 94%, respectively. As shown in Figure 9, greater enhancement of the peak area of BPA can be observed apparently after the extraction by the resulting composite. The average enrichment factor of BPA (*n* = 3) was calculated to be 4.9 respectively. In fact, the enrichment factor of the resulting composite can be improved further by increasing the volume of sample because of its high adsorption efficiency (>90%) for low levels of BPA. Moreover, nine adsorption-desorption cycles were repeated to evaluate the reusability of Fe_3_O_4_@CTS/SiO_2_-C_12_ composite through using 10 mg/L BPA solution. The adsorption rate of BPA was kept at 92 ± 4% ($\bar{x}$ ± SD, *n* = 9). The adsorption efficiency of the resulting composite has no obvious change after reusing nine times. Therefore, Fe_3_O_4_@CTS/SiO_2_-C_12_ composite can be proposed as an easily recyclable and efficient adsorbent for the enrichment of trace BPs from water. 

## 4. Conclusions

In this work, magnetic dodecyl chitosan/silica composite was developed and employed as an efficient adsorbent for the effective enrichment of BPs from aqueous solution. The modification of the dodecyl group can increase significantly the interaction between the resulting composite and BPs. The hydrophobic interaction was proposed as the principal mechanism for the adsorption of BPs over magnetic dodecyl chitosan/silica composite. In addition, hydrogen-bonding interaction also plays a role in the adsorption of BPs. Kinetic and isotherm studies showed that the multilayer adsorption behavior carried out on the heterogeneous surface of the composite. The reusability of the resulting composite can be quickly achieved by little solvent washing and magnetic separation technologies. Magnetic dodecyl chitosan/silica composite showed facile reusability and high enrichment efficiency for the enrichment of BPs from water. Magnetic dodecyl chitosan/silica composite has a potential to be applied in the preconcentration of other trace organic pollutants including BPs in aqueous sample. 

## Figures and Tables

**Table 1:** Kinetic parameters of the adsorption data of BPA on Fe_3_O_4_@CTS/SiO_2_-C_12_ composite.

*C*_0_ (mg/L)	Pseudo-First Order		Pseudo-Second Order	
	*k*_1_ (min^−1^)	*R* ^2^	*k*_2_ (g·mg^−1^·min^−1^)	*R* ^2^
10	0.0868	0.8439	0.6828	0.9986
20	0.2095	0.8796	0.4495	0.9998
40	0.2319	0.9149	0.3386	0.9999
50	0.1858	0.9630	0.1838	0.9999

**Table 2:** Parameters of adsorption isotherm models.

Langmuir Isotherm			Freundlich Isotherm		
*q_m_*	*K_L_*	*R* ^2^	1/*n*	*InK_F_*	*R* ^2^
2.188	0.8064	0.9792	0.8236	0.515	0.9947
